# Awareness and Understanding of Post-intensive Care Syndrome (PICS) Among Internal Medicine Residents in a Community Hospital Setting

**DOI:** 10.7759/cureus.108014

**Published:** 2026-04-30

**Authors:** Shihla Shireen Kanamgode, Sharmitha Ravichandran, Arunava Saha, Zeeshan Gulam Hussain, Kevin Martin

**Affiliations:** 1 Pulmonology and Critical Care, Umass Memorial Medical Center, Worcester, USA; 2 Internal Medicine, Saint Vincent Hospital, Worcester, USA; 3 Pulmonary and Critical Care Medicine, Tulane University School of Medicine, Worcester, USA; 4 Hospital Medicine, Saint Vincent Hospital, Worcester, USA; 5 Pulmonary and Critical Care Medicine, Saint Vincent Hospital, Worcester, USA

**Keywords:** cognitive impairment, critical illness recovery, educational gaps, knowledge assessment, long-term icu complications awareness, physical impairment, post-intensive care syndrome (pics), psychological sequelae, questionnaire-based study, resident education

## Abstract

Background

Post-intensive care syndrome (PICS) includes cognitive, psychological, and physical impairments that may persist after critical illness. Prior studies have shown that more than half of ICU survivors develop new physical, mental, or cognitive problems after discharge, underscoring the substantial burden of post-ICU morbidity. Despite this, awareness among healthcare providers remains limited. Internal medicine residents frequently care for ICU survivors during hospitalization and after discharge, and limited awareness may delay recognition of post-ICU complications and contribute to gaps in continuity of care.

Objective

To assess awareness of PICS, knowledge of its clinical domains and management concepts, and self-reported confidence in managing post-ICU sequelae among internal medicine residents in a community hospital and identify educational gaps.

Methods

We conducted a cross-sectional electronic survey of internal medicine residents at a community-based residency program from June to July 2023. The questionnaire assessed knowledge of PICS, familiarity with the Assess, prevent, and manage pain; Both spontaneous awakening trials and spontaneous breathing trials; Choice of analgesia and sedation; Delirium: assess, prevent, and manage; Early mobility and exercise; Family engagement and empowerment (ABCDEF) bundle, prior PICS education, and confidence in managing post-ICU sequelae. Descriptive statistics were used to summarize responses. Exploratory associations between prior PICS education and selected outcomes were assessed using Fisher's exact test, with odds ratios, 95% confidence intervals, and two-sided P values reported.

Results

Sixty-five of 75 residents responded (86.7%). Residents demonstrated substantial knowledge gaps regarding PICS, particularly in identifying affected populations, diagnostic tools, and familiarity with the ABCDEF bundle. Prior PICS education was significantly associated with greater familiarity with the ABCDEF bundle (OR, 11.47; 95% CI, 1.03-601.36; P = .023) and higher confidence in managing post-ICU sequelae (OR, 6.71; 95% CI, 1.71-33.17; P = .004), but not with other knowledge measures. These exploratory associations should be interpreted with caution, as the wide confidence intervals suggest limited precision and possible instability of some estimates.

Conclusions

Internal medicine residents demonstrated substantial knowledge gaps regarding PICS. Targeted educational strategies during residency may strengthen recognition and management of post-ICU complications.

## Introduction

Annually, over 5.7 million individuals are admitted to intensive care units (ICUs) across the globe, with nearly 4.8 million emerging as survivors of their critical conditions [1]. However, the journey to recovery extends far beyond ICU discharge. A substantial proportion of ICU survivors, exceeding 50% according to observational studies, may experience a constellation of persistent health problems collectively referred to as post-intensive care syndrome (PICS) [2]. This syndrome includes impairments in cognitive, physical, and mental health domains and can significantly affect the long-term well-being of both patients and their families [3,4]. Post-intensive care syndrome was formally described in the critical care literature in 2012 following stakeholder efforts to define the long-term consequences of critical illness [4]. Since its formal description in the critical care literature, awareness of PICS has gradually increased over the past decade as ICU survivorship has improved and greater attention has been directed toward the long-term physical, cognitive, and psychological sequelae of critical illness [3].

PICS is highly prevalent among survivors of critical illness. A prospective multicenter study found that 58% of medical ICU survivors, 64% of urgent surgical ICU survivors, and 43% of elective surgical ICU survivors developed new physical, mental, or cognitive problems one year after ICU discharge [5]. Another meta-analysis of 15 prospective and four retrospective cohort studies, including 10,179 patients, reported a pooled prevalence of PICS of 54.35% (95% CI, 45.54%-63.15%) within the first 12 months after ICU discharge. The highest prevalence was observed in studies from South America and North America, reaching 61.95% (95% CI, 28.33%-95.62%) [6]. Importantly, these impairments can persist long after the initial critical illness. Several studies have demonstrated that physical, cognitive, psychiatric, and quality-of-life deficits may continue for five years or longer following ICU discharge and may result in permanent disability in some patients [7,8].

Insights into the profound impact of PICS come from pivotal studies such as the Bringing to Light the Risk Factors and Incidence of Neuropsychological Dysfunction in ICU Survivors (BRAIN-ICU) study, a multicenter prospective cohort investigation of 821 patients who survived shock or respiratory failure requiring mechanical ventilation [9]. Follow-up assessments revealed that nearly 40% of survivors had cognitive deficits comparable to moderate traumatic brain injury at three months, while 26% exhibited mild dementia-like impairments at 12 months. Physical disability was also common, with 32% experiencing difficulty with basic daily activities at three months and 26% reporting challenges with more complex tasks. In addition, 37% of survivors reported symptoms of depression [9].

Management of PICS requires a multidisciplinary approach involving cognitive assessment tools such as the Montreal Cognitive Assessment (MoCA) [10], mental health screening instruments including the Patient Health Questionnaire-9 (PHQ-9) [11], and evaluations by physical and occupational therapists [12]. Preventive and management strategies emphasize patient-centered ICU care practices, including minimizing sedation, promoting early mobilization, and encouraging family engagement during critical illness [13].

Internal medicine residents play an important role in the care of ICU survivors. They participate in patient management during ICU admission, continue care after transfer to medical wards, and often encounter these patients again in outpatient settings after hospital discharge. In many situations, residents may be the first clinicians to evaluate patients experiencing post-ICU sequelae. Therefore, a strong understanding of PICS is essential for early recognition, prevention, and management. Given the high prevalence and long-term impact of PICS, limited awareness among residents may delay recognition of post-ICU complications and contribute to gaps in continuity of care. This study aims to assess internal medicine residents’ awareness of PICS as a clinical entity, knowledge of its domains, affected populations, diagnostic approaches, and prevention strategies, and confidence in managing post-ICU sequelae, in order to identify educational gaps and opportunities for improving residency education.

## Materials and methods

This study aimed to assess the awareness and understanding of post-intensive care syndrome (PICS) among internal medicine residents in a community hospital. A structured questionnaire was developed by the research team to evaluate multiple aspects of PICS knowledge and awareness; the full survey questionnaire is provided in Appendix 1. The survey evaluated awareness of PICS, knowledge of its core clinical features and prevention strategies, and self-reported confidence in managing post-ICU sequelae. Items were developed based on relevant PICS literature and expert review, with questions designed to assess key domains of the syndrome, including affected domains, susceptible populations, diagnostic tools, prevention strategies, and follow-up concepts. The questionnaire was reviewed by critical care faculty members for content relevance and clarity prior to distribution. The instrument was not pilot tested and did not undergo formal psychometric validation or reliability assessment, including Cronbach’s alpha; accordingly, it should be considered nonvalidated. The study protocol was submitted to the institutional review board (IRB), which determined that the project was exempt from review because it did not involve patient data or human subjects.

The survey consisted of 10 questions addressing key domains related to PICS, including domains affected by PICS, susceptible populations, diagnostic tools, prevention strategies, familiarity with the Assess, prevent, and manage pain; Both spontaneous awakening trials and spontaneous breathing trials; Choice of analgesia and sedation; Delirium: assess, prevent, and manage; Early mobility and exercise; Family engagement and empowerment (ABCDEF) bundle, prior education regarding PICS, sources of education, confidence in managing post-ICU sequelae, perceived impact of PICS education on patient care, and duration of PICS symptoms after ICU discharge. The questionnaire was distributed to residents through a Google Forms (Google LLC, Mountain View, California, USA) link, and participation was encouraged through bi-weekly reminders over a period of 1.5 months from June 2023 to July 2023. Responses were collected anonymously. A total of 65 responses were obtained from 75 residents.

All internal medicine residents in the program were eligible for participation. Because the study was designed as a cross-sectional survey of the entire accessible resident cohort at a single institution, a formal a priori sample size calculation was not performed. Instead, all eligible residents were invited to participate to maximize response capture and minimize sampling bias.

The collected data were entered into Microsoft Excel (Microsoft Corporation, Redmond, Washington, USA) for analysis. Descriptive statistics were used to summarize survey responses, and results were reported as frequencies and percentages. For exploratory analyses, associations between prior PICS education and selected categorical outcomes were assessed using Fisher exact test. These outcomes included correct identification of the domains affected by PICS, recognition that both patients and family members may be affected, correct identification of diagnostic tools, correct identification of prevention strategies, familiarity with the ABCDEF bundle, and confidence in managing post-ICU sequelae.

For these exploratory analyses, selected survey responses were dichotomized to permit two-group comparisons. Confidence in managing post-ICU sequelae was categorized as confident (very confident or somewhat confident but would like to learn more) versus not confident (needed more information). Familiarity with the ABCDEF bundle was categorized as familiar versus not familiar or unsure. Odds ratios (ORs), 95% confidence intervals (CIs), and two-sided P values were reported, and a P value of less than .05 was considered statistically significant.

## Results

**Figure 1:**
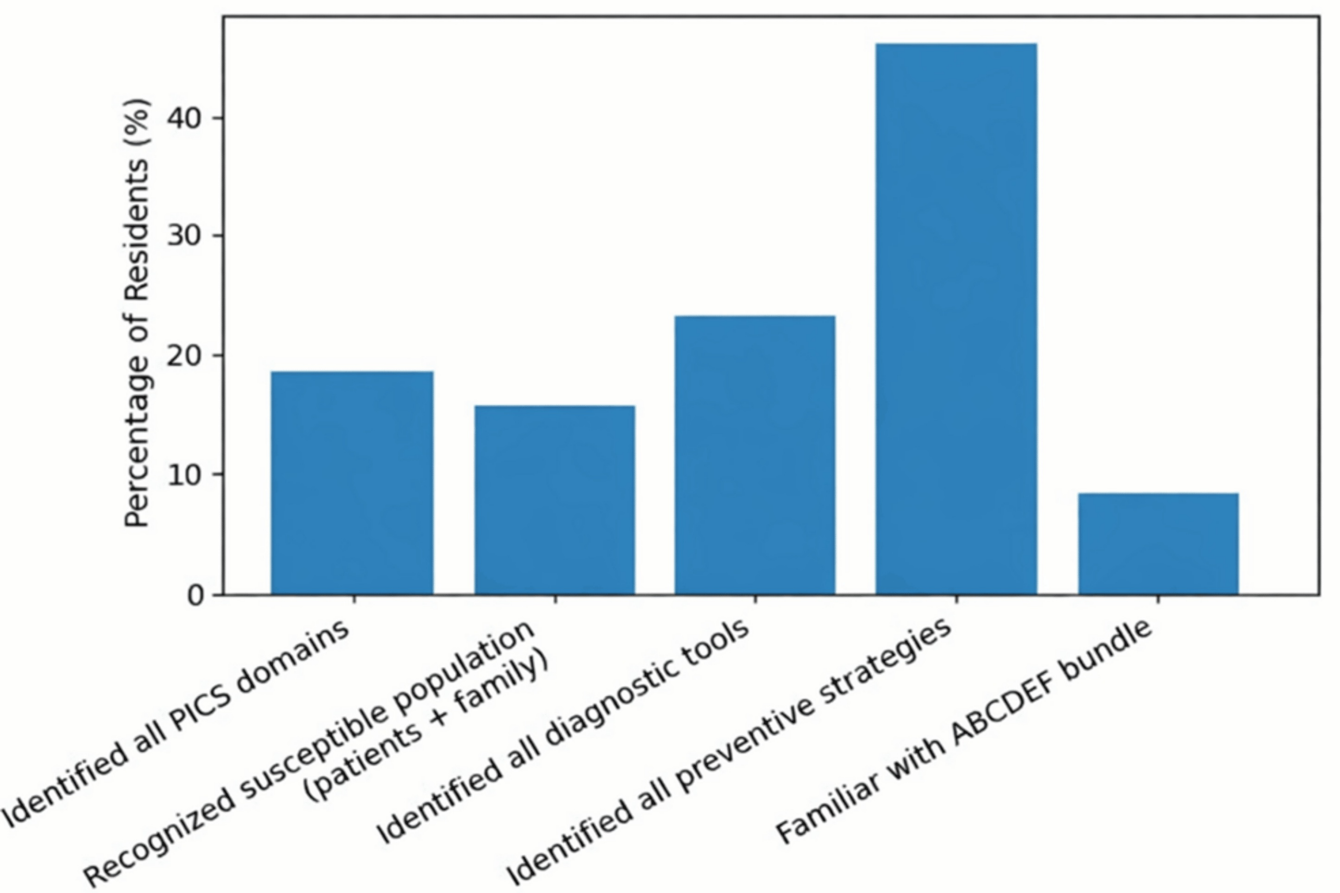
Key Knowledge Indicators of Post-intensive Care Syndrome (PICS) Among Residents. ABCDEF: Assess, prevent, and manage pain; Both spontaneous awakening trials and spontaneous breathing trials; Choice of analgesia and sedation; Delirium: assess, prevent, and manage; Early mobility and exercise; Family engagement and empowerment.

Among the 65 residents surveyed, overall awareness of post-intensive care syndrome (PICS) was limited (Table 1, Figure 1). Fewer than one-quarter of residents correctly identified all key domains of PICS, recognized that both patients and family members may be affected, or identified all recommended diagnostic tools. Awareness of the ABCDEF bundle was particularly poor, with only five residents (7.7%) reporting familiarity. In contrast, knowledge of some individual preventive strategies was better, and 30 residents (46.2%) correctly identified all recommended prevention measures.

**Table 1:** Residents’ Knowledge and Awareness of Post-Intensive Care Syndrome (PICS) (N = 65). MoCA: Montreal Cognitive Assessment, PHQ: Patient Health Questionnaire, PT: physical therapy, H&P: history and physical examination, ABCDEF: Assess, prevent, and manage pain; Both spontaneous awakening trials and spontaneous breathing trials; Choice of analgesia and sedation; Delirium: assess, prevent, and manage; Early mobility and exercise; Family engagement and empowerment.

Variable	Response	n (%)
Understanding of PICS domains	Correctly identified all domains (psychiatric, physical, cognitive)	12 (18.5)
Perception of susceptible population	Patients and family members (correct choice)	10 (15.4)
	Patients only	34 (52.3)
	Patients and doctors	21 (32.3)
Recognition of diagnostic tools for PICS	Correctly identified all tools (MoCA, PT evaluation, PHQ-2 [14], PHQ-9, H&P)	15 (23.1)
Preventive strategies for PICS	Correctly identified all strategies	30 (46.2)
	Delirium monitoring and management	52 (80.0)
	Early mobilization	56 (86.2)
	Family empowerment and engagement	53 (81.5)
	Daily sedative interruption/management	42 (64.6)
	Breathing trials	34 (52.3)
Familiarity with ABCDEF bundle	Familiar	5 (7.7)
	Unsure	9 (13.8)
	Not familiar	51 (78.5)

Residents' attitudes, confidence, and perceptions regarding PICS are summarized in Table 2. Formal education on PICS was limited, with only 19 residents (29.2%) reporting prior education within the past five years. Confidence in managing post-ICU sequelae was also generally low, with 28 residents (43.1%) categorized as confident and 37 (56.9%) categorized as not confident. Despite these gaps, nearly all residents agreed that improving knowledge of PICS would enhance patient care, and most recognized that PICS symptoms may persist for weeks, months, or longer after ICU discharge.

**Table 2:** Residents’ Attitudes, Confidence, and Perceptions Regarding PICS (N = 65). *Percentages calculated among respondents who reported prior education on PICS (n = 19). PICS: post-intensive care syndrome, USMLE: United States Medical Licensing Examination.

Variable	Response	n (%)
Previous education on PICS (past 5 years)	Yes	19 (29.2)
	No	46 (70.8)
Sources of PICS education*	USMLE study materials	9 (48.6)
	Residency program	7 (37.1)
	Medical school	2 (8.6)
	Self-study	1 (2.9)
Confidence in managing post-ICU sequelae	Very confident	1 (1.5)
	Somewhat confident but want more information	27 (41.5)
	Need more information	37 (56.9)
Impact of learning more about PICS on patient care	Strongly agree	47 (72.3)
	Somewhat agree	16 (24.6)
	Disagree	1 (1.5)
Perceived duration of PICS symptoms	Weeks to months/years	58 (89.2)

Exploratory analyses examining the association between prior PICS education and selected outcomes are presented in Table 3. Prior PICS education was not significantly associated with correct identification of PICS domains, recognition that both patients and family members may be affected, correct identification of diagnostic tools, or correct identification of prevention strategies. However, prior PICS education was significantly associated with greater familiarity with the ABCDEF bundle (OR, 11.47; 95% CI, 1.03-601.36; Fisher exact P = .023) and higher confidence in managing post-ICU sequelae (OR, 6.71; 95% CI, 1.71-33.17; Fisher exact P = .004).

**Table 3:** Association Between Prior PICS Education on Knowledge and Confidence Outcomes. PICS: post-intensive care syndrome, ABCDEF: Assess, prevent, and manage pain; Both spontaneous awakening trials and spontaneous breathing trials; Choice of analgesia and sedation; Delirium: assess, prevent, and manage; Early mobility and exercise; Family engagement and empowerment.

Outcome	Odds ratio	95% CI	P value
Correct identification of PICS domains	0.89	0.14-4.37	1.00
Correct recognition that both patients and family members may be affected	0.79	0.07-5.01	1.00
Correct identification of diagnostic tools	2.08	0.51-8.11	.33
Correct identification of prevention strategies	2.63	0.78-9.50	.10
Familiarity with the ABCDEF bundle	11.47	1.03-601.36	.023
Confidence in managing post-ICU sequelae	6.71	1.71-33.17	.004

## Discussion

This study evaluated awareness and understanding of post-intensive care syndrome (PICS) among internal medicine residents at a community hospital and identified substantial gaps in knowledge. A large proportion of residents demonstrated limited understanding of the multidimensional nature of PICS and the populations it affects. Many respondents incorrectly believed that PICS affects only patients, despite growing evidence that family members may also experience psychological and emotional consequences following critical illness. Many respondents incorrectly believed that PICS affects only patients, despite growing evidence that family members may also experience psychological and emotional consequences following critical illness. This misconception may reflect limited formal teaching on PICS-family during residency, as training is often centered on the patient’s acute medical course rather than the broader long-term impact of critical illness on families. In addition, residents may have limited exposure to structured post-ICU follow-up care, where family-related sequelae are more likely to be recognized. This is important because PICS-family extends beyond psychological distress alone and may also include physical and socioeconomic burdens that can adversely affect both family well-being and the patient’s recovery [15]. Recognition of diagnostic approaches was also incomplete. Although many residents identified the role of physical therapy in assessing functional impairment, fewer than one-quarter were able to recognize the full range of recommended diagnostic tools used in evaluating PICS. In addition, familiarity with the ABCDEF bundle, a widely recommended framework designed to improve ICU care and reduce long-term complications, was extremely limited. The ABCDEF bundle includes assessment and management of pain, spontaneous awakening and breathing trials, choice of analgesia and sedation, delirium assessment and prevention, early mobility, and family engagement [16]. Low awareness among residents may reflect variable ICU exposure and limited formal teaching on recovery-oriented ICU care. Prior work has similarly shown that most residents were initially unfamiliar with the bundle, with significant improvement in knowledge after a targeted multimodal educational intervention [17]. Together, these findings suggest that important aspects of post-ICU recovery remain underrecognized during residency training.

Many prior studies have reported similar findings. A quantitative cross-sectional observational pilot study involving 51 critical care physicians, nurses, nurse practitioners, and physician assistants at a university hospital demonstrated that knowledge of PICS, including its symptoms and risk factors, was generally low. Confidence in recognizing the condition was also limited, with no significant differences observed across clinician roles [18]. Similarly, another study including 382 healthcare providers, comprising 205 nurses, 126 attending physicians, and 51 advanced practice providers, reported inconsistent awareness of PICS among clinicians involved in the care of critically ill patients [19]. Comparable barriers have also been identified in primary care, where physicians reported limited awareness of PICS and PICS-family terminology, inadequate education of patients and families, and time constraints in caring for survivors of critical illness [20]. Our findings are consistent with these reports and further show that awareness gaps are also substantial among internal medicine residents, with fewer than one-quarter of respondents correctly identifying all PICS domains, affected populations, or diagnostic tools, and only 7.7% reporting familiarity with the ABCDEF bundle. This is also supported by a recent resident-focused educational study in which most trainees initially reported poor baseline familiarity with the ABCDEF bundle, with significant improvement in knowledge after a multimodal teaching intervention [21]. Together, these findings suggest that gaps in knowledge regarding PICS and related recovery frameworks extend beyond trainees and intensivists and may affect multiple disciplines involved in the longitudinal care of ICU survivors.

Limited exposure to structured education on PICS may contribute to reduced confidence in managing post-ICU sequelae. Many residents reported requiring additional information to effectively care for ICU survivors and believed that increased education on PICS would improve patient care. Variability in perceptions regarding the duration of PICS symptoms also suggests uncertainty regarding the long-term course of the syndrome, which may influence follow-up planning and continuity of care for ICU survivors. Improving awareness of PICS among trainees may facilitate earlier recognition of cognitive, psychological, and functional impairments following critical illness and allow timely referral for rehabilitation and supportive services.

Exploratory analyses provided additional insight into the relationship between prior education and resident preparedness. In our study, prior PICS education was significantly associated with greater familiarity with the ABCDEF bundle and higher self-reported confidence in managing post-ICU sequelae, but not with correct identification of PICS domains, susceptible populations, diagnostic tools, or prevention strategies. This pattern suggests that prior educational exposure may improve awareness of practical recovery frameworks and perceived readiness for care more readily than detailed content knowledge across all domains. These findings should be interpreted with caution, however, as the wide confidence intervals, particularly for ABCDEF bundle familiarity, indicate limited precision and possible instability of some estimates, likely reflecting the small sample size and low frequency of certain outcomes.

Educational interventions may help address these gaps. In a pilot study of a virtual interprofessional training module for internal medicine residents, participants demonstrated significant improvement in objective knowledge scores and confidence related to recovery after critical illness following structured PICS-focused teaching. Mean knowledge scores improved from 51% before the course to 79% after the course, and confidence in facilitating discussions about recovery from critical illness also increased significantly. These findings suggest that deficiencies in PICS awareness and preparedness may be modifiable through targeted educational interventions, supporting the incorporation of structured PICS teaching into residency curricula [22].

The gaps identified in our study are particularly important in light of the broader literature describing the burden and complexity of PICS. A recent narrative review emphasized that PICS consists of new or worsening physical, cognitive, and psychological impairments after critical illness and that these sequelae may persist well beyond hospital discharge. The review also highlighted the importance of structured assessment and multidisciplinary follow-up after ICU discharge and noted that even clinicians outside dedicated PICS clinics should incorporate elements of PICS evaluation into routine practice whenever feasible. These observations reinforce the clinical importance of improving resident awareness, as internal medicine residents frequently participate in transitions of care and may be among the first clinicians to evaluate ICU survivors after discharge [23].

Although specialized ICU recovery clinics have been developed to address the long-term needs of ICU survivors, their availability remains limited. Currently, only about 35 dedicated ICU follow-up clinics operate in the United States [24]. As a result, awareness and recognition of PICS must extend beyond intensivists to clinicians at all levels of training. In many cases, early identification and management of post-ICU sequelae will occur in general inpatient or outpatient settings, where internal medicine residents and primary care providers often serve as the first point of contact for patients after ICU discharge.

This study has several strengths. The survey achieved a high response rate among residents, strengthening the reliability of the findings and reducing the likelihood of nonresponse bias. In addition, the study assessed multiple aspects of PICS awareness, providing a broad overview of residents’ understanding of the condition and highlighting key educational gaps. The inclusion of exploratory comparative analyses also added depth to the findings by identifying specific areas in which prior education was associated with improved awareness and confidence.

Several limitations should be acknowledged. Because this was a single-center study conducted at a community-based residency program, the findings may not be generalizable to academic, tertiary-care, or international settings, where resident exposure to critical care, post-ICU follow-up, and formal education on PICS may differ. The questionnaire was developed specifically for this study and was not pilot tested or formally validated, including reliability testing, and therefore should be considered nonvalidated. This may have introduced measurement bias and affected the internal validity of the study. In addition, the results were based on self-reported knowledge and perceptions and may not fully reflect residents’ actual clinical practice or decision-making. The study was not designed with a formal priori sample size calculation and may have been underpowered to detect smaller associations across some knowledge domains. Accordingly, some exploratory estimates were imprecise, as reflected by wide confidence intervals that may indicate limited precision and possible instability of effect estimates. The survey also did not assess differences in knowledge based on prior ICU exposure in detail, and prior educational exposure was evaluated only in a limited manner, both of which may have influenced awareness of PICS.

Based on these findings, several educational interventions were implemented within our institution to address the identified knowledge gaps. These initiatives included targeted lectures, small-group discussions, and educational posters focusing on PICS and post-ICU recovery. In addition, PICS education has now been incorporated into the routine curriculum for internal medicine residents to improve awareness and preparedness in managing the long-term consequences of critical illness.

## Conclusions

This study identified substantial gaps in awareness and understanding of post-intensive care syndrome among internal medicine residents in a community hospital setting. Despite the high prevalence of PICS among ICU survivors and its important long-term effects on patients and their families, many residents demonstrated limited knowledge of its domains, affected populations, diagnostic approaches, and preventive strategies. Awareness of key frameworks such as the ABCDEF bundle was particularly limited, and many residents reported low confidence in managing post-ICU sequelae. These findings support the need for structured educational initiatives to strengthen resident preparedness in addressing the long-term consequences of critical illness.

Incorporating PICS-focused education into residency training may enhance clinicians’ ability to recognize post-ICU complications, support earlier intervention, and strengthen continuity of care for ICU survivors and their families. Potential strategies include structured didactic modules, case-based discussions, integration of PICS concepts into ICU and ambulatory curricula, and exposure to post-ICU recovery or follow-up care models where feasible.

## Appendices

Appendix 1

**Table 4:** Survey Instrument Administered in this Study. Credits: Shihla Shireen Kanamgode, Sharmitha Ravichandran, Arunava Saha, Zeeshan Gulam Hussain, and Kevin Martin. This questionnaire was developed by the authors for the present study. PHQ: Patient Health Questionnaire, USMLE: United States Medical Licensing Examination, ABCDEF: Assess, prevent, and manage pain; Both spontaneous awakening trials and spontaneous breathing trials; Choice of analgesia and sedation; Delirium: assess, prevent, and manage; Early mobility and exercise; Family engagement and empowerment.

Item	Question	Response instruction	Response options
	Participant information		
	You are invited to participate in an anonymous survey designed to assess medical residents’ knowledge and opinions regarding Post-Intensive Care Syndrome (PICS). No personal information will be collected. Participation is voluntary, and you may skip any question or stop the survey at any time without any impact on your residency. Completion of the survey implies consent to participate.	Informational statement	Not applicable
	Knowledge of PICS		
1	Which of the following domains are affected by Post-Intensive Care Syndrome (PICS)?	Check all that apply	Breathing function; Psychiatric function; Physical function; Cognitive function
2	In your opinion, who is susceptible to PICS?	Check all that apply	Doctors; Patients; Patients' family members; Nurses
3	Which of the following are diagnostic tools for PICS?	Check all that apply	Montreal Cognitive Assessment (MoCA); Physical therapy evaluation; PHQ-2/PHQ-9 evaluation; History and examination
4	Which of the following may help prevent PICS?	Check all that apply	Daily sedative interruption; Early mobilization; Breathing trial; Delirium monitoring and management; Family empowerment and engagement
5	Are you familiar with the ABCDEF bundle approach for prevention and management of PICS?	Mark only one option	Yes; No; Not sure
	Education and confidence		
6	Have you received prior education about PICS within the past 5 years?	Mark only one option	Yes; No
7	If yes, where did you receive education about PICS?	Mark only one option; if applicable, specify other	Medical school; USMLE study materials; Residency program; Other: ______
8	How confident do you feel managing post-ICU sequelae in the outpatient setting?	Mark only one option; if applicable, specify other	Very confident; Somewhat confident but would like to learn more about PICS; I need more information; Other: ______
	Perceptions of clinical relevance		
9	Do you think learning about PICS would help improve patient care?	Mark only one option; if applicable, specify other	Strongly agree; Somewhat agree; Disagree; Other: ______
10	How long do you think PICS lasts after discharge from the ICU?	Mark only one option	Less than 6 weeks; 6 weeks to 6 months; 6 months to 1 year; Usually more than 1 year

## References

[1] Weissman GE, Kerlin MP, Yuan Y, Gabler NB, Groeneveld PW, Werner RM, Halpern SD (2018). Population trends in intensive care unit admissions in the United States among Medicare beneficiaries, 2006-2015. Ann Intern Med.

[2] Griffiths J, Hatch RA, Bishop J, Morgan K, Jenkinson C, Cuthbertson BH, Brett SJ (2013). An exploration of social and economic outcome and associated health-related quality of life after critical illness in general intensive care unit survivors: a 12-month follow-up study. Crit Care.

[3] Inoue S, Nakanishi N, Amaya F (2024). Post-intensive care syndrome: recent advances and future directions. Acute Med Surg.

[4] Needham DM, Davidson J, Cohen H (2012). Improving long-term outcomes after discharge from intensive care unit: report from a stakeholders' conference. Crit Care Med.

[5] Geense WW, Zegers M, Peters MA (2021). New physical, mental, and cognitive problems 1 year after ICU admission: a prospective multicenter study. Am J Respir Crit Care Med.

[6] Tilburgs B, Simons KS, Corsten S (2025). Associations between physical, cognitive, and mental health domains of post-intensive care syndrome and quality of life: a longitudinal multicenter cohort study. Crit Care Med.

[7] Ayenew T, Gete M, Gedfew M (2025). Prevalence of post-intensive care syndrome among intensive care unit-survivors and its association with intensive care unit length of stay: systematic review and meta-analysis. PLoS One.

[8] Herridge MS, Tansey CM, Matté A (2011). Functional disability 5 years after acute respiratory distress syndrome. N Engl J Med.

[9] Bienvenu OJ, Friedman LA, Colantuoni E (2018). Psychiatric symptoms after acute respiratory distress syndrome: a 5-year longitudinal study. Intensive Care Med.

[10] Nasreddine ZS, Phillips NA, Bédirian V (2005). The Montreal Cognitive Assessment, MoCA: a brief screening tool for mild cognitive impairment. J Am Geriatr Soc.

[11] Kroenke K, Spitzer RL (2002). The PHQ-9: a new depression diagnostic and severity measure. Psychiatr Ann.

[12] Pandharipande PP, Girard TD, Jackson JC (2013). Long-term cognitive impairment after critical illness. N Engl J Med.

[13] Mikkelsen ME, Still M, Anderson BJ (2020). Society of Critical Care Medicine's international consensus conference on prediction and identification of long-term impairments after critical illness. Crit Care Med.

[14] Kroenke K, Spitzer RL, Williams JB (2003). The Patient Health Questionnaire-2: validity of a two-item depression screener. Med Care.

[15] Shirasaki K, Hifumi T, Nakanishi N (2024). Postintensive care syndrome family: a comprehensive review. Acute Med Surg.

[16] Marra A, Ely EW, Pandharipande PP, Patel MB (2017). The ABCDEF bundle in critical care. Crit Care Clin.

[17] Garcia P, Quiñones Cruz KM, Ramsaroop T, Acosta Sánchez I, Ascoli C, Ferrer Marrero TM (2025). Improving resident physicians' knowledge of the ABCDEF bundle through a multimodal teaching intervention. Int J Med Educ.

[18] Sepúlveda P, Gallardo A, Arriagada R, González E, Rocco PR, Battaglini D (2025). Protocolized strategies to encourage early mobilization of critical care patients: challenges and success. Crit Care Sci.

[19] Lobos PG, Nairon EB, Denbow M, Olson DM, Wilson JE (2024). Critical care clinicians' knowledge of post-intensive care syndrome. AACN Adv Crit Care.

[20] Rolfsen ML, Mart MF, Kieffer H (2026). Post-intensive care syndrome awareness and communication: surveys of ICU providers and patients. Chest.

[21] Liou A, Schweickert WD, Files DC, Bakhru RN (2023). A survey to assess primary care physician awareness of complications following critical illness. J Intensive Care Med.

[22] Hampton SF, Carlbom D, Steinkruger S (2022). Interprofessional education module on post-intensive care syndrome for internal medicine residents. ATS Sch.

[23] Schwitzer E, Jensen KS, Brinkman L (2023). Survival ≠ recovery: a narrative review of post-intensive care syndrome. Chest Crit Care.

[24] Butcher BW (2026). Post-intensive care syndrome. JAMA.

